# Whole-genome sequencing for surveillance of fluoroquinolone resistance in rifampicin-susceptible tuberculosis in a rural district of Shanghai: A 10-year retrospective study

**DOI:** 10.3389/fpubh.2022.990894

**Published:** 2022-09-15

**Authors:** Yangyi Zhang, Yuan Jiang, Chenlei Yu, Jing Li, Xuhui Shen, Qichao Pan, Xin Shen

**Affiliations:** ^1^Division of Tuberculosis and HIV/AIDS Prevention, Shanghai Municipal Center for Disease Control and Prevention, Shanghai, China; ^2^Shanghai Institutes of Preventive Medicine, Shanghai, China; ^3^Department of Epidemiology, School of Public Health and Key Laboratory of Public Health Safety, Fudan University, Shanghai, China

**Keywords:** fluoroquinolone resistance, whole-genome sequencing, rifampicin-susceptible tuberculosis, tuberculosis surveillance, hetero-resistance

## Abstract

**Background:**

Fluoroquinolones (FQs) are the most important second-line anti-tuberculosis (anti-TB) drugs, primarily used for the treatment of multidrug- or rifampicin-resistant TB (MDR/RR-TB). However, FQs are also commonly used to treat other bacterial infections. There are few published data on the rates of FQ resistance among rifampicin-susceptible TB.

**Methods:**

We used whole-genome sequencing (WGS) to determine the prevalence of FQ resistance among rifampicin-susceptible TB in a rural district of Shanghai. This was a population-based retrospective study of all culture-positive pulmonary TB patients diagnosed in the Chongming district of Shanghai, China during 2009–2018.

**Results:**

The rate of FQ resistance was 8.4% (29/345) among TB, 6.2% (20/324) among rifampicin-susceptible TB, and 42.9% (9/21) among MDR/RR-TB. Transmission of FQ-resistant strains was defined as strains differing within 12 single-nucleotide polymorphisms (SNPs) based on WGS. Among the rifampicin-susceptible TB, 20% (4/20) of FQ resistance was caused by the transmission of FQ-resistant strains and 45% (9/20) of FQ resistance was identified as hetero-resistance.

**Conclusions:**

The prevalence of FQ resistance in rifampicin-susceptible TB was higher than expected in Shanghai. Both the transmission and the selection of drug-resistant strains drive the emergence of FQ resistance in rifampicin-susceptible TB isolates. Therefore, the WGS-based surveillance system for TB should be urgently established and the clinical awareness of the rational use of FQs for respiratory infections should be enhanced to prevent the premature occurrence of FQ resistance.

## Introduction

Fluoroquinolones (FQs) are the most important second-line anti-tuberculosis drugs. The World Health Organization (WHO) recommended moxifloxacin and levofloxacin as Group A agents for use in multidrug- or rifampicin-resistant tuberculosis (MDR/RR-TB) regimens because they can significantly reduce the risk of treatment failure or relapse and death in MDR/RR-TB (1). Moxifloxacin, a fourth-generation FQ, is currently being considered by the WHO for use in four-month regimens for drug-susceptible tuberculosis (TB) because of its excellent pharmacokinetics and drug-penetration into macrophages (2, 3). Thus, in addition to being the cornerstone of the regimens for MDR/RR-TB, FQs will be the key drugs in the shorter regimen for drug-susceptible TB in the future.

According to the technical specifications on TB prevention and control in China, FQs are still mainly used for the treatment of MDR/RR-TB. To date, few studies have investigated the prevalence of FQ resistance among rifampicin-susceptible TB, and the majority of these investigations indicate that FQ resistance in rifampicin-susceptible TB is uncommon (4, 5). However, FQs are a class of broad-spectrum antibiotics that differ from other anti-TB drugs, which are widely used to treat other bacterial infections, especially respiratory infections (6). In addition, several studies have shown that FQs have been extensively used in health facilities for the diagnostic treatment of patients with suspected TB and for the empirical treatment of TB patients without a drug susceptibility testing (DST) result (7, 8). The inappropriate use and the vital role of FQs in TB treatment bring our attention to the premature development of FQ resistance in rifampicin-susceptible *Mycobacterial tuberculosis* (MTB) isolates in TB high-burden settings.

Whole-genome sequencing (WGS) is promising to be an ideal tool for the surveillance of drug resistance in TB (9). Furthermore, WGS can offer information on hetero-resistance, resulting in more precise predictions of drug resistance phenotypes (10). Mutations in the *gyrA* and *gyrB* genes, which code for two subunits of DNA gyrase, have been identified as the main causes of FQ resistance in MTB (10, 11). The issue of hetero-resistance appears to be more frequent in FQ resistance (12, 13). Multiple large comparative studies have demonstrated that the accuracy of WGS in predicting phenotypic resistance to rifampicin, isoniazid, and FQs is high. The several databases of high-confidence resistance-conferring variants have also been developed (13–15).

In this study, we present the results from a WGS-based retrospective study of all MTB isolates from cases of pulmonary TB diagnosed in the Chongming district of Shanghai, China during 2009–2018. We aimed to determine the prevalence of FQ resistance, particularly among rifampicin-susceptible TB. Additionally, we identified the hetero-resistance in FQ resistance and quantified the FQ resistance due to the transmission of FQ-resistant strains.

## Materials and methods

### Study design and participants

This was a retrospective study that included all the culture-positive pulmonary TB patients who were reported by local designated hospitals in the Chongming district of Shanghai, China, between Jan 1, 2009, and Dec 31, 2018. According to the TB surveillance system in Shanghai, all local inhabitants aged 15 years or older with TB symptoms were referred to local TB designated hospitals for diagnosis, which involved the use of sputum smear and Lowenstein-Jensen (L-J) medium culture. All clinical isolates of pulmonary TB patients were submitted to Shanghai Municipal Center for Disease Control and Prevention (Shanghai CDC) TB reference laboratory for species identification and strain preservation. Demographic, clinical, and microbiological records were obtained from the national TB information management system.

### WGS and bioinformatics analysis

Stored isolates were revived on L-J medium and inactivated in a water bath of 80°C in a biosafety laboratory. Genomic DNA was extracted and purified using the QIAamp DNA Mini Kit (Qiagen, Hilden, GER) and sequenced on the Hiseq 2500 platform (Illumina, San Diego, CA, USA) with an expected coverage of 100. Raw sequencing data was trimmed and filtered using fastp v0.23.1 (16). Paired-end reads were mapped to the reference genome H37Rv (GenBank NC_000962.3) with BWA-MEM v0.7.17. Genetic variants, including SNPs and small insertions/deletions (indels), were called using SAMtools v1.6 and BCFtools v1.6 (17).

For WGS-based DST, WGS predictions of drug resistance phenotypes to drugs were based on a list of drug resistance-conferring mutations (13). The following 16 anti-TB drugs were tested: rifampicin, fluoroquinolones, isoniazid, ethambutol, pyrazinamide, streptomycin, ethionamide, amikacin, capreomycin, kanamycin, para-aminosalicylic acid, cycloserine, linezolid, bedaquiline, clofazimine, and delamanid. The allele frequency threshold of 10% was used to predict resistance. Hetero-resistance was defined based on the frequency of resistant alleles in the sequence reads <99% in this study.

To assess the transmission of FQ resistance, the fixed SNPs (frequency ≥ 75%), which were not in drug resistance-conferring mutations nor in PPE/PE-PGRS family genes (18), were used to calculate the pairwise SNP distances between FQ-resistant isolates. Clusters of isolates potentially consistent with recent transmission were identified using the genomic threshold of ≤ 12 SNPs (19). The strain lineages/sub-lineages were identified according to the SNP schemes previously established (20, 21).

A maximum likelihood (ML) phylogenetic tree of FQ-resistant isolates was inferred using RAxML-NG v1.0.2 (22) with the GTR+GAMMA model of nucleotide substitution and 100 bootstraps. The phylogenetic tree was visualized and annotated with iTol (https://itol.embl.de/).

### Definitions of primary and acquired FQ resistance

Both primary and acquired FQ resistance were determined by the time of diagnosis, FQ resistance-conferring mutations, and genomic cluster. The primary FQ resistance (transmitted FQ resistance) was defined as the FQ resistance-conferring mutation shared by more than or equal to two strains in a genomic cluster with the removal of the FQ resistance-conferring mutation of the earliest onset strain among them; the remaining FQ resistance-conferring mutations were considered to be acquired FQ resistance.

### Statistical analysis

All statistical analyses were done using IBM SPSS v.20 software. The Pearson's chi-squared or Fisher's exact test was used for comparison of categorical variables, such as demographic, bacteriological, and clinical characteristics. Ages were presented as the mean with a standard deviation. The Mann-Whitney *U*-test was used for the comparison of ages. A *P* < 0.05 was defined as significant.

## Results

### Characteristics of the patients and MTB isolates

Totally, 370 culture-positive pulmonary TB patients who were reported in the Chongming district between 2009 and 2018 were enrolled in this study. Of these patients, 25 (6.8%) were excluded from analysis due to strain contamination, recovery failure, or WGS failure. Among the remaining 345 patients, 283 (82%) were male, 297 (86.1%) were new TB cases, and the average age was 56 years (range 17 to 93 years). According to the results of WGS-based DST, 21 (6.1%) were diagnosed with MDR/RR-TB. MDR/RR-TB patients were more likely to have been previously treated for TB (47.6 vs. 11.7%; *p* < 0·0001) than rifampicin-susceptible TB patients (Table 1).

**Table 1:** Characteristics of MDR/RR-TB and rifampicin-susceptible TB patients in Chongming, Shanghai^α^.

	**MDR/RR-TB** **(*n* = 21)**	**Rifampicin-susceptible TB**	***p***
		**(*n* = 324)**	
**Demographic factors**			
Age (yr), mean ± SD	46 ± 20	57 ± 21	0.018^*^
Gender			0.391
Female	2 (9.5)	60 (18.5)	
Male	19 (90.5)	264 (81.5)	
Census register			1.000
Resident	19 (90.5)	285 (88.0)	
Migrant	2 (9.5)	39 (12.0)	
**Clinical factors**			
Case detection			0.106
Referral	16 (76.2)	186 (57.4)	
Clinical consultation	4 (19.0)	129 (39.8)	
Physical examination	1 (4.8)	9 (2.8)	
TB treatment history			<0.0001^*^
No	11 (52.4)	286 (88.3)	
Yes	10 (47.6)	38 (11.7)	
Pulmonary cavity			0.556
No	15 (71.4)	211 (65.1)	
Yes	6 (28.6)	113 (34.9)	
Positive sputum smear result			0.630
No	5 (23.8)	93 (8.7)	
Yes	16 (76.2)	231 (91.3)	
**Bacteriological factors**			
Genomic clustered			0.249
No	13 (93.1)	238 (73.5)	
Yes	8 (6.9)	86 (26.5)	
lineage			0.201
Non-Beijing	2 (9.5)	45 (13.9)	
Ancient Beijing	8 (38.1)	67 (20.7)	
Modern Beijing	11 (52.4)	212 (65.4)	

^α^Data are *n* (%); MDR, multidrug resistant; RR, rifampicin resistant; TB, tuberculosis.

* *P* < 0.05 is statistically significant.

### Prevalence and mutation types of FQ resistance

Across the 345 isolates tested for WGS-based DST, 29 (8.4%) were resistant to FQ, including 20 rifampicin-susceptible isolates and 9 rifampicin-resistant isolates. The rate of FQ resistance was 6.2% (20/324) among rifampicin-susceptible TB and 42.9% (9/21) among MDR/RR-TB. The drug resistance profile and epidemiological information of 29 FQ-resistant TB patients were shown in the Supplementary Table S1.

On sequence analysis, nine rifampicin-resistant isolates harbored the FQ resistance-conferring mutations in the *gyrA* gene, including four *gyrA* D94A and five *gyrA* A90V. Seventeen rifampicin-susceptible isolates harbored diverse FQ resistance-conferring mutations, including nine *gyrA* D94G, four *gyrA* D94A, two *gyrA* D94Y, one *gyrA* A90V, and one *gyrA* S91P. In addition, three rifampicin-susceptible isolates harbored more than one FQ resistance-conferring mutation, including *gyrA* A90V+S91P+D94A, *gyrA* S91P+D94N, and *gyrA* D94G+*gyrB* T500N.

**Figure 1:**
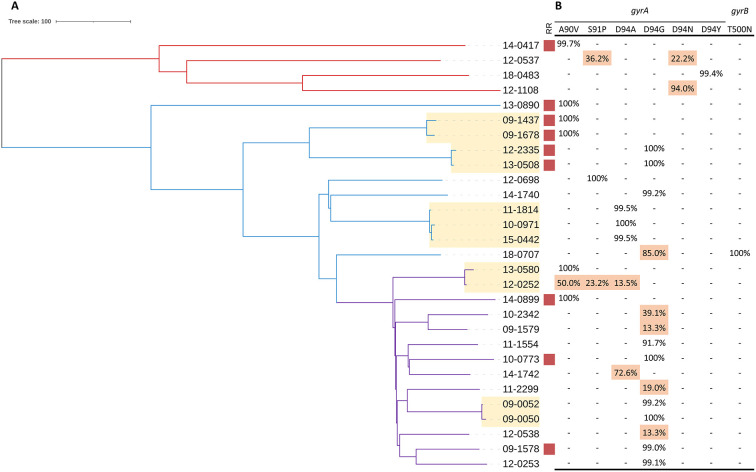
Phylogeny, clustering and FQ hetero-resistance profile of 29 FQ-resistant MTB isolates. **(A)** Red, blue and purple branches indicate Lineage 4, ancient Beijing and modern Beijing strains, respectively. Genomic-clustered strains differing by ≤ 12 SNPs are highlighted in yellow. Red square indicates the MTB isolate resistant to rifampicin. **(B)** Allele frequency of FQ resistance-conferring loci in *gyrA* and *gyrB*. Hetero-resistance mutations are highlighted in red.

On allele frequency of drug resistance-conferring mutations, FQ hetero-resistance was only observed in the rifampicin-susceptible isolates. Among 20 rifampicin-susceptible isolates with FQ resistance-conferring mutations, 9 were identified as FQ hetero-resistance. The allele frequencies of FQ hetero-resistance ranged from 13.3 to 94.0%. Of the FQ hetero-resistant isolates, seven had a single unfixed mutation and two had multiple unfixed mutations in *gyrA*. The allele frequencies of FQ resistance-conferring mutations in 29 FQ-resistant isolates were shown in Figure 1B.

### Transmission of FQ-resistant isolates

A total of 11 FQ-resistant isolates were grouped into 5 genomic clusters defined as strains that differed by 12 or fewer SNPs, among which 7 were rifampicin-susceptible isolates and 4 were rifampicin-resistant isolates. Drug resistance mainly emerges in two ways: acquired drug resistance due to inadequate therapy, or primary drug resistance caused by the transmission of drug-resistant strains. Among the 11 FQ-resistant clustered isolates, all the paired strains carried the identical FQ resistance-conferring mutations except for one, which carried three unfixed mutations. Notably, we were able to observe the frequency dynamics of FQ resistance-conferring mutations in this recent transmission cluster (patient 12-0252 and patient 13-0580). Patient 12-0252 was diagnosed in 2012 with unfixed FQ resistance-conferring mutations (*gyrA* 50% A90V + 23.2% S91P + 13.5% D94A); the *gyrA* A90V mutation became fixed during the subsequent infection of patient 13-0580 (Figure 1). By comparing the transmission and resistance-conferring mutations of FQ-resistant isolates, we found that the FQ resistance of 20% (4/20) of rifampicin-susceptible isolates and 22.2% (2/9) of rifampicin-resistant isolates resulted from the transmission of FQ-resistant strains.

## Discussion

The WGS of MTB is rapidly developing from being a scientific research tool to a drug resistance and epidemiological surveillance tool of TB for public health protection (9, 23). In the current study, we used WGS to retrospectively investigate the prevalence of FQ resistance and its transmission in all MTB isolates from pulmonary TB patients diagnosed in Chongming, Shanghai for 10 years. Our results showed that rifampicin-susceptible TB accounted for most (69%) of the cases of FQ-resistant TB overall. The prevalence of FQ resistance among rifampicin-susceptible TB was 6.1%, a high proportion (45%) of which was identified as hetero-resistance. By WGS analysis, the transmission of FQ-resistant strains resulted in 20% of FQ resistance in rifampicin-susceptible TB.

In our study population, more than two-thirds of the FQ resistance was detected in rifampicin-susceptible TB patients. It is well known that FQs are the core agents for MDR/RR-TB treatment. In China, moxifloxacin and levofloxacin are usually prescribed only to MDR/RR-TB patients. This prescription practice aligns with the technical specifications on TB prevention and control. As a result, FQ DST is not routinely performed on rifampicin-susceptible TB patients, and there are few surveillance data on the prevalence of FQ resistance in rifampicin-susceptible TB. The present study found that among rifampicin-susceptible TB patients in Shanghai, the prevalence of FQ resistance in MTB clinical isolates was higher (6.1%) than that reported in other countries, ranging from 0 to 4.4% (4, 5, 24, 25). China is one of the high TB burden countries, with a large number of active TB cases and latent TB infections. In addition, FQs are the most commonly prescribed antibiotics for respiratory infections in Shanghai (26). There is therefore a need to conduct surveillance of FQ resistance and FQ exposure in newly diagnosed TB patients in high TB burden countries.

Previous studies have shown that *gyrA* D94G and A90V are the most prevalent FQ resistance-conferring mutations, which have been reported to confer higher-level FQ resistance or lower fitness cost *in vitro* (27–32). All of the FQ resistance-conferring mutations occurring in rifampicin-resistant MTB in this study were D94G or A90V, suggesting that MTB carrying multiple other drug resistance-conferring mutations might be more likely to acquire FQ resistance-conferring mutations with a lower fitness cost. The patterns of FQ resistance-conferring mutations occurring in rifampicin-susceptible MTB showed more diversity. All the FQ hetero-resistance was detected in rifampicin-susceptible MTB. Hetero-resistance is a crucial phase in the progression of an originally drug-susceptible MTB population becoming completely drug-resistant to a given drug during the course of an infection (32). Non-lethal drug concentration facilitates the emergence of drug mutations and the selection of mutations with a low fitness cost (33, 34). Based on these hypotheses, the features of the FQ resistance-conferring mutation in rifampicin-susceptible MTB imply that the FQ resistance in rifampicin-susceptible MTB might be induced by inefficient FQ therapy. Several studies have demonstrated that the proportion of TB patients exposed to FQs before TB diagnosis is high, due to the easy access and inappropriate use of FQs (7, 8, 35–37). Devasia et al. reported that more than 10 days of FQ exposure prior to TB diagnosis is a primary risk factor for FQ resistance (38). Meanwhile, MTB rapidly acquires FQ resistance during moxifloxacin monotherapy (39). In recent years, with the use of large-scale genotype-phenotype analyses, substantial improvements have been made in the correlation of genotype with resistance phenotype (9, 15). However, hetero-resistance limits the ability to detect drug resistance of rapid molecular assays (12). The variant allele frequencies provided by WGS could be used to identify hetero-resistance for better predictions of drug resistance phenotypes (10, 11). FQ hetero-resistance was found in nine rifampicin-susceptible MTB isolates in our investigation, with hetero-resistance frequencies ranging from 13.3 to 94.0% as identified by WGS. These findings have two important implications. First, WGS makes the detection of FQ resistance more sensitive, especially in hetero-resistant strains. Second, the real burden of FQ resistance in rifampicin-susceptible TB might be underestimated.

Transmission of drug-resistant strains is a major driver of the high prevalence of drug-resistant TB in China (19, 40). In this study, over 20% of FQ resistance was regarded as primary drug resistance caused by the transmission of FQ-resistant strains. The rates of primary drug resistance of FQ in rifampicin-susceptible and rifampicin-resistant MTB isolates were similar. Due to the massive migrant population in Shanghai and inadequate therapy, almost one-third of MDR-TB cases were attributed to recent transmission (19). Transmission of rifampicin-resistant strains also facilitated the spread of FQ resistance in this study. One unanticipated finding was that a similar proportion of FQ resistance caused by transmission was observed in rifampicin-susceptible MTB isolates. A meta-analysis of nine studies concluded that empirical FQ prescriptions for respiratory infections are linked to delays in pulmonary TB diagnosis and treatment (35), and such delays might contribute to the transmission of TB (41). Intriguingly, our results displayed the transition of multiple unfixed FQ resistance-conferring mutations to a single fixed mutation during transmission, which suggests that *gyrA* A90V is likely a FQ resistance-conferring mutation with a low fitness cost *in vivo*.

Our study was limited by the retrospective study design so that the TB patients' information on FQ prescriptions prior to diagnosis, such as date, dosage, type of FQ, and days of supply, could not be obtained in this study. A prospective cohort study is warranted to further understand the source of FQ resistance in rifampicin-susceptible TB.

In summary, the prevalence of FQ resistance among rifampicin-susceptible TB was 6.1% in Shanghai, which was more than expected. The results of WGS analysis showed that half of the FQ resistance in rifampicin-susceptible TB was hetero-resistance and that the transmission of FQ-resistant strains also contributed to the emergence of FQ-resistant TB. Therefore, the WGS-based surveillance system for TB should be urgently established and the clinical awareness of the rational use of FQs for respiratory infections should be enhanced to prevent the premature occurrence of FQ resistance.

## Data availability statement

The datasets presented in this study can be found online at: https://www.ncbi.nlm.nih.gov/bioproject/?term=PRJNA760838.

## Ethics statement

The studies involving human participants were reviewed and approved by the Ethics Committee of the Shanghai Municipal Center for Disease Control and Prevention (No. 2020-14). Written informed consent for participation was not required for this study in accordance with the national legislation and the institutional requirements.

## Author contributions

YZ and XiS designed the study, drafted, and revised the manuscript. CY, JL, and XuS did the laboratory work. YZ did the data analyses. YJ, QP, and XiS supervised the project. All authors contributed to the article and approved the submitted version.

## Funding

This work was supported by the National Natural Science Foundation of China (Grant No. 81872679), Science and Technology Innovation Plan of Shanghai Science and Technology Commission (Grant No. 21DZ2202400), Shanghai Municipal Project for Academic Leaders in Health, Three-Year Action Plan of Shanghai Public Health System Construction (Grant No. GWV-10.1-XK03), and Key Young Talents Training Program for Shanghai Disease Control and Prevention (Grant No. 21QNGG01).

## Conflict of interest

The authors declare that the research was conducted in the absence of any commercial or financial relationships that could be construed as a potential conflict of interest.

## Publisher's note

All claims expressed in this article are solely those of the authors and do not necessarily represent those of their affiliated organizations, or those of the publisher, the editors and the reviewers. Any product that may be evaluated in this article, or claim that may be made by its manufacturer, is not guaranteed or endorsed by the publisher.
